# The association between early diagnosis of gestational diabetes and maternal-neonatal outcomes: a secondary analysis of the digest trial

**DOI:** 10.1007/s12020-025-04542-y

**Published:** 2026-02-16

**Authors:** Sarah Dib, Nan Luo, Danielle L Jones, Suzanne Smith, Roy Taylor, Helen R Murphy, Laura C Kusinski, Claire L Meek

**Affiliations:** 1 Leicester Diabetes Centre and Leicester NIHR Biomedical Research Centre, University of Leicester, Leicester General Hospital, Gwendoline Road, Leicester, LE5 4PW UK; 2 Institute of Metabolic Science – Medical Research Laboratories, Cambridge Biomedical Campus, University of Cambridge, Hills Road, Cambridge, CB2 0QQ UK; 3 Magnetic Resonance Centre, Newcastle University, Campus for Ageing and Vitality, Newcastle upon Tyne, Newcastle, NE4 5PL UK; 4 University of East Anglia, Bob Champion Building, Research Park, Rosalind Franklin Rd, Norwich, NR4 7UQ UK; 5 Cambridge University Hospitals NHS Foundation Trust, Cambridge Biomedical Campus, Hills Road, Cambridge, UK; 6 University Hospitals of Leicester NHS Trust, Leicester General Hospital, Gwendoline Road, Leicester, LE5 4PW UK

**Keywords:** Gestational diabetes, Early gestational diabetes, Timing of diagnosis, Neonatal outcomes, Weight management, Dietary intervention

## Abstract

**Purpose:**

To determine whether early gestational diabetes (GDM) differs from later GDM in maternal characteristics and perinatal outcomes.

**Methods:**

This is a secondary analysis of an energy-restricted dietary intervention in GDM (DiGest) randomized controlled trial. We compared maternal weight, glycemia, and pregnancy/neonatal outcomes between the early GDM (< 20 weeks, *n* = 118) and standard GDM diagnosis (21–28 weeks, *n* = 299) groups.

**Results:**

Early GDM was associated with higher antenatal (40 vs. 38 mmol/mol; *p* = 0.017) and postnatal HbA1c (38 vs. 36 mmol/mol; *p* = 0.002) and higher risk of diabetes/prediabetes postnatally (19 vs. 6%; *p* = 0.003). Despite higher medication requirements, perinatal outcomes did not differ. Lower gestational weight gain (2.5 vs. 5.3 kg, *p* = 0.003) and comparable glycemia at 36 weeks were found. Timing of diagnosis did not impact the effect of the DiGest intervention.

**Conclusion:**

Early GDM reflects more severe underlying hyperglycemia, but timely treatment and reduced gestational weight gain can offset adverse perinatal risks.

**Supplementary Information:**

The online version contains supplementary material available at 10.1007/s12020-025-04542-y.

## Background

It is currently recommended to screen for gestational diabetes mellitus (GDM) at 24–28 weeks of gestation followed by treatment to reduce the risk of adverse outcomes [1, 2]. However, rising prevalences of maternal comorbidities such as obesity raises concerns that some pregnant women may develop GDM before 24 weeks, suggesting that earlier screening may be beneficial. Previous studies have shown that an early diagnosis of GDM, or even marginal elevations of first-trimester fasting glucose levels, is associated with adverse pregnancy outcomes [3–6]. The results of Treatment of Booking Gestational Diabetes Mellitus (TOBOGM) trial demonstrated a reduced risk of adverse neonatal outcomes [7] and improved breastfeeding initiation [8] with early pregnancy treatment of GDM (< 20 weeks) as compared with deferred treatment.

From a pathophysiological perspective, there are two possible explanations for early GDM. Firstly, early GDM could represent a more severe version of GDM, with an early hyperglycemic response to pregnancy hormones rising at 20 weeks [9, 10]. An alternative option would be that early and standard GDM represent two distinct phenotypes of diabetes first identified in pregnancy, consistent with undiagnosed pre-pregnancy glucose intolerance for early GDM, rather than a response to placental hormones that rise at 20 weeks which manifests as GDM during routine screening.

The aim of this analysis was to compare the characteristics, pregnancy and neonatal outcomes and treatment strategies of women diagnosed early (< 20 weeks) vs. later (21–28 weeks) in participants recruited as part of the Dietary Intervention in Gestational Diabetes (DiGest) trial [11]. We also aimed to compare the effects of the DiGest trial intervention on the primary outcomes (maternal weight change and infant birth weight) according to timing of diagnosis.

## Research design and methods

### Study Design

This is a secondary analysis using data collected as part of the DiGest trial, a multicenter randomized controlled double-blind trial involving 425 participants with gestational diabetes. Participants were randomized to receive a standard-energy (*n* = 211; 2000 kcal/day) or reduced-energy (*n* = 214; 1200 kcal/day) dietbox from 29 weeks until delivery. The primary outcomes were maternal weight change between 28 and 36 weeks gestation and infant birth weight. The protocol, eligibility criteria and primary results are published elsewhere [11, 12]. The trial (ISRCTN: 65152174) was ethically approved by the UK Research Ethics Committee (Reference 18/WM/0191) and the National Health Service (NHS) Health Research Authority (IRAS 242924). Informed consent was obtained from all individual participants included in the study.

### Participants and Diagnosis

Participants were eligible if they were diagnosed with gestational diabetes before 30 + 6 weeks’ gestation, had a pre-pregnancy BMI ≥ 25 kg/m^2^, and had a singleton pregnancy. The diagnosis of gestational diabetes was based on the criteria of the National Institute for Health and Care Excellence (NICE) [2]: 75 g OGTT ≥ 5.6 mmol/L (≥ 100 mg/dL) fasting and ≥ 7.8 mmol/L (≥ 140 mg/dL) at 2 h; with glucometer testing recurrently above targets of ≥ 5.3 mmol/L fasting (≥ 95 mg/dL) and ≥ 7.8 mmol/L (≥ 140 mg/dL) 1 h after meal in those with previous gestational diabetes who declined OGTT. During the COVID-19 pandemic (2020–2022), the diagnostic criteria were expanded to include the Royal College of Obstetricians and Gynaecologists interim COVID-19 criteria (random glucose 9–11 mmol/L equivalent to 162–198 mg/dL or HbA1c 41–47 mmol/mol at booking; fasting glucose ≥ 5.6 mmol/L equivalent to ≥ 100 mg/dL or HbA1c ≥ 39 mmol/mol at 28 weeks gestation) [13]. Women who have had GDM in a previous pregnancy were offered an OGTT as soon as possible after booking. Early diagnosis was defined as women who had a diagnosis of GDM before 20^+ 6^ weeks of gestation whereas standard diagnosis was between 21 and 28 weeks gestation.

All study centres followed NICE guidelines for treatment of GDM, offering a period of dietary change followed by metformin and/or insulin for women with persistent hyperglycemia above targets set by NICE. Treatment did not differ between those diagnosed early vs. later, except in duration where an early diagnosis triggered the early initiation of the clinical care pathway of GDM. Diet and physical activity at baseline are described elsewhere [14].

### Clinical Measures

Visits were arranged at 28–30 weeks (baseline), 32 weeks (V2) and 36 weeks (V3) of gestation and at ~ 3 months postnatally (V4) when relevant maternal and neonatal data were collected.

Maternal clinical measures included in this analysis are glycemic measures assessed using a masked Dexcom G6 continuous glucose monitor (CGM) worn for 7–10 days at baseline, 36 weeks and 3 months postpartum; HbA1c percentage at baseline and 3 months postpartum; maternal weight and BMI at baseline, 36 weeks and 3 months postpartum; gestational weight gain estimated by subtracting weight at booking from weight at 36 weeks; and treatments administered for GDM at 28–30 weeks. Information about maternal demographics including ethnicity, age, deprivation according to Index of Multiple Deprivation (IMD), and education was also collected.

Pregnancy and neonatal outcomes including type of delivery, gestational age, birthweight z-score (using INTERGROWTH-21st ), large/small-for-gestational age (LGA; SGA), admission to the neonatal intensive care unit (NICU), and incidence of neonatal hypoglycemia (< 46.7 mg/dL within the first 48 h of life) were collected from medical records. Infant feeding (any breastfeeding, exclusive breastfeeding) was assessed at 3 months postpartum with any breastfeeding defined as receiving any breast milk and exclusive breastfeeding as receiving no formula milk (with no information about solid foods or other liquids).

### Statistical analysis

Analysis was completed using SPSS v.28.0. Descriptive results are presented as mean ± SD or n (%). The difference between groups at baseline was assessed by t-test or Chi-square test as appropriate. To investigate the association between timing of diagnosis (early vs. later) and maternal/pregnancy/neonatal outcomes, univariate linear and logistic regression models were used. These models were adjusted for trial arm and study center as confounders. Models for the association between timing of diagnosis and coefficient of variation (CV) and standard deviation (SD) were additionally adjusted for average CGM glucose. To investigate whether timing of diagnosis influenced the effects of the DiGest dietary intervention on the primary maternal (maternal weight change 36 weeks-baseline) and neonatal (birthweight z-score) outcomes, linear regression adjusted for timing of diagnosis (early vs. standard) and study center was used. P-values < 0.05 were considered statistically significant.

## Results

### Baseline characteristics of early vs. Standard diagnosis groups

417/425 participants had data on timing of diagnosis and were included in this analysis. Out of 417, 118 were diagnosed < 20 weeks (28%) at an average of 13–14 weeks (Sup. Table 1). Participants diagnosed early were less likely to be primiparous (17.5% vs. 43.9%, *p* < 0.001), more likely to smoke or vape (17.1% vs. 8.4%, *p* = 0.011), and more likely to have had GDM before (57.3% vs. 16.4%, *p* < 0.001). They also had a significantly higher BMI at ~ 12 weeks (at booking) but gained significantly less weight between booking and 28–29 weeks (2.17 ± 5.62 vs. 4.80 ± 5.93 kg, *p* < 0.001). At 28–29 weeks, participants diagnosed early had significantly higher HbA1c concentrations (40.12 ± 4.19 vs. 38.27 ± 4.77 mmol/mol; *p* = 0.017) and were more likely to be using metformin and short- and long-acting insulin. There were no significant differences between the two groups in any maternal demographics or CGM metrics at 28–29 weeks.

## Pregnancy and neonatal outcomes

There were no significant differences in any pregnancy, delivery, neonatal or breastfeeding outcomes (Table 1).

**Table 1:** The association between timing of diagnosis and maternal, pregnancy and neonatal outcomes

	*n*	Early diagnosis (*n* = 118)	*n*	Standard diagnosis (*n* = 299)	Mean Difference or Odds Ratio (95%CI)	*p*-value
Maternal Outcomes:						
Glycaemia at 36 Weeks (V3)						
**Mean CGM glucose mg/dL**	63	105.87 ± 15.35	159	103.71 ± 14.08	2.09 (-2.17, 6.36)	0.335
**TIR** **(63–120 mg/dL)%**	63	73.17 ± 18.58	159	76.26 ± 18.41	-3.00 (-8.45, 2.45)	0.279
**TAR** **(63–120 mg/dL)%**	63	25.01 ± 19.76	159	22.04 ± 19.21	2.89 (-2.83, 8.61)	0.321
**TBR** **(63–120 mg/dL)%**	63	1.81 ± 3.14	159	1.70 ± 3.14	0.11 (-0.81, 1.04)	0.814
**TIR** **(63–140 mg/dL)%**	63	88.45 ± 11.85	159	90.64 ± 10.27	-2.18 (-5.36, 0.99)	0.176
**TAR** **(63–140 mg/dL)%**	63	9.74 ± 12.41	159	7.67 ± 10.49	2.07 (-1.20, 5.34)	0.213
**TBR** **(63–140 mg/dL)%**	63	1.81 ± 3.14	159	1.70 ± 3.14	0.11 (-0.81, 1.04)	0.814
**SD**	63	1.12 ± 0.34	159	1.05 ± 0.30	0.06 (-0.02, 0.13)	0.142
**CV**	63	19.08 ± 4.49	159	18.16 ± 4.37	0.95 (-0.35, 2.24)	0.150
**Glycaemia at ~ 3 Months Postpartum (V4)**						
**Mean CGM glucose mg/dL**	53	116.09 ± 16.18	141	111.84 ± 12.20	4.52 (0.24, 8.80)	**0.039**
**TIR (63–120 mg/dL)%**	53	59.67 ± 23.86	141	66.94 ± 19.33	-7.59 (-14.20, -0.98)	**0.025**
**TAR (63–120 mg/dL)%**	53	39.64 ± 24.50	141	32.38 ± 19.88	7.66 (0.86, 14.45)	**0.027**
**TBR (63–120 mg/dL)%**	53	0.69 ± 1.98	141	0.68 ± 1.53	-0.07 (-0.60, 0.46)	0.805
**TIR (63–140 mg/dL)%**	53	83.59 ± 17.05	141	88.31 ± 10.81	-4.80 (-8.91, -0.70)	**0.022**
**TAR (63–140 mg/dL)%**	53	15.71 ± 17.42	141	11.01 ± 11.08	4.87 (0.68, 9.07)	**0.023**
**TBR (63–140 mg/dL)%**	53	0.69 ± 1.98	141	0.68 ± 1.53	-0.07 (-0.60, 0.46)	0.805
**TIR (70–180 mg/dL)%**	53	96.72 ± 6.41	141	97.49 ± 4.27	-0.63 (-2.22, 0.96)	0.436
**TAR (70–180 mg/dL)%**	53	1.92 ± 5.58	141	0.96 ± 2.67	0.95 (-0.23, 2.14)	0.114
**TBR (70–180 mg/dL)%**	53	1.36 ± 3.83	141	1.55 ± 3.65	-0.33 (-1.51, 0.86)	0.588
**SD**	53	1.07 ± 0.28	141	1.05 ± 0.21	-0.01 (-0.08, 0.05)	0.731
**CV**	53	16.64 ± 3.42	141	16.92 ± 3.16	-0.21 (-1.26, 0.85)	0.700
**HbA1c mmol/mol**	70	37.99 ± 3.98	175	36.27 ± 3.92	1.73 (0.62, 2.83)	**0.002**
**Weight**						
**36-week BMI kg/m2**	109	36.60 ± 6.55	245	35.52 ± 6.20	1.17 (-0.25, 2.57)	0.105
**Postnatal BMI kg/m2**	72	33.47 ± 6.47	185	32.51 ± 6.06	1.17(-0.53, 2.86)	0.177
**36 weeks (V3)- 29 weeks (Baseline) weight change (kg)**	112	0.38 ± 4.33	268	0.48 ± 4.20	-0.09 (-1.02, 0.85)	0.853
**Estimated total gestational weight gain (36 weeks - booking; kg)**	112	2.50 ± 6.54	267	5.30 ± 6.83	-2.68 (-4.15, -1.21)	**< 0.001**
**Risk of prediabetes/diabetes postnatally**	70	13 (18.6)	175	10 (5.7)	3.91 (1.60, 9.57)	**0.003**
**Neonatal and Pregnancy Outcomes**:						
**Cesarean Section**	**118**	42 (35.6)	**299**	133 (44.5)	1.49 (0.95, 2.32)	0.081
**Pre-eclampsia**	**114**	1 (0.9)	**261**	6 (2.3)	2.75 (0.33, 23.25)	0.353
**Hypertension**	**114**	4 (3.5)	**261**	15 (5.7)	1.72 (0.56, 5.32)	0.345
**Birthweight (g)**	**114**	3287.36 ± 483.90	**261**	3294.83 ± 470.03	-3.02 (-108.20, 102.16)	0.955
**Birthweight z-score**	**113**	0.54 ± 1.02	**261**	0.44 ± 0.94	0.12 (-0.10, 0.33)	0.282
**Gestational week at birth**	114	38.24 ± 1.22	262	38.52 ± 1.32	-0.28 (-0.57, 0.00)	0.052
**SGA**	113	2 (1.8)	261	13 (5.0)	3.02 (0.67, 13.66)	0.152
**LGA**	113	22 (19.5)	261	52 (19.9)	1.01 (0.58, 1.76)	0.975
**NICU admission**	114	9 (7.9)	260	29 (11.2)	1.44 (0.66, 3.16)	0.360
**Neonatal hypoglycemia**	114	4 (3.5)	260	15 (5.8)	1.66 (0.53, 5.16)	0.386
**Neonatal jaundice**	114	12 (10.5)	260	29 (11.2)	1.07 (0.52, 2.19)	0.852
**Infant feeding at 3 months**						
**Receiving any breastmilk**	79	49 (62.0)	192	111 (57.8)	0.89 (0.51, 1.53)	0.661
**Receiving breast milk only**	79	32 (40.5)	192	72 (37.5)	0.90 (0.53, 1.55)	0.716

Linear or logistic regression models adjusted for trial assignment and study center were used to determine odds ration/mean difference (95% CI). Models for the association between timing of diagnosis and CV and SD were additionally adjusted for average CGM glucose. Birthweight z-score was adjusted for gestation at birth and sex using INTERGROWTH birthweight standard CGM: continuous glucose monitoring; TIR: time in range; TAR: time above range; TBR: time below range; SD: standard deviation; CV: coefficient of variation; BMI: body mass index; SGA: small for gestational age infant, birthweight centile < 10; LGA: large for gestational age infant, birthweight centile > = 90; NICU: neonatal intensive care unit. Baseline (V1): 28–30 weeks gestation; Visit 3: 36 weeks gestation; Visit 4: 3 months postpartum. 63–120 mg/dL is equivalent to 3.5–6.7 mmol/L whereas 63–140 mg/dL is equivalent to 3.5–7.8 mmol/L and 70–140 mg/dL to 3.9–10.0 mmol/L. Risk of pre-diabetes/diabetes postnatally is defined as having HbA1c > 41 mmol/mol

### Maternal outcomes (Glycaemia and Weight)

At ~ 3 months postnatally (V4), participants diagnosed early had significantly higher average CGM glucose and HbA1c concentrations and spent significantly less time in range (63–140 mg/dL & 63–120 mg/dL) and more time above range (Table 1). A higher percentage of participants diagnosed early than at standard time were at risk of diabetes/pre-diabetes (HbA1c > 41 mmol/mol). There were no significant differences in maternal BMI at 36 weeks, BMI at 3 months, or in weight change during the DiGest intervention; however, participants diagnosed early had a significantly lower average gestational weight gain (weight at 36 weeks - weight at booking).

### Intervention effects x timing of diagnosis

Adjusting for timing of diagnosis did not influence the effect of the intervention on the primary maternal outcome (weight change) and neonatal outcome (birthweight) (Sup. Table 2).

## Conclusions

Early diagnosis (< 20 weeks) of GDM was associated with more severe hyperglycemia on HbA1c and higher risk of prediabetes/diabetes postnatally. However, we did not find an increased risk of adverse pregnancy or neonatal outcomes in the early diagnosis group, or any significant differences in weight or glycemic measures during pregnancy suggesting that early intervention through medication and reduced gestational weight gain may mitigate perinatal risks.

### Strengths and limitations

The main strength of our study is the collection of detailed clinical data, particularly CGM data at different phases during pregnancy and postnatally as part of a double-blind, randomized controlled trial. However, since this secondary analysis is based on observational data, the possibility of confounding remains despite adjusting for pre-specified potential confounders. Additionally, it is possible that this secondary study was not powered to detect true differences in certain clinical outcomes. This also meant that corrections for multiple testing were not made to not reduce statistical power further. Another limitation is that the GDM diagnosis method differed among participants particularly due to the Covid-19 pandemic which necessitated changes to the diagnostic criteria. Lastly, all participants had a BMI ≥ 25 kg/m^2^ which can limit generalizability of the findings to women with GDM who are not classified as overweight or obese.

### Results in relation to previous literature

Previous studies showed higher risk of adverse pregnancy and neonatal outcomes with early GDM diagnosis, despite earlier treatment; although these risks were often attenuated after adjusting for maternal factors like adiposity and dysglycemia [4, 5]. Simmons et al. [15] also demonstrated adverse perinatal outcomes with early GDM diagnosis (< 20 weeks) despite treatment from 24 to 28 weeks. In contrast, we found no significant increase in adverse perinatal outcomes between early and standard diagnosis groups. The major difference between the present study and previous observational studies of early GDM is that women were cared for in centers involved with the DiGest study with likely greater focus upon dietary control of total energy intake. It is likely that this accounts for the improvement in the early GDM group such as to bring about comparable maternal BMI and CGM metrics later in pregnancy. The effect of higher medication use in the early GDM group will also have contributed although this is unlikely to explain differences from previous observational studies. This aligns with the TOBOGM trial, which supported early treatment of GDM (< 20 weeks) over deferred treatment [7], reiterating that timely and appropriate dietary and pharmaceutical management may mitigate early GDM-related risks.

Despite similar glycemia and weight in mid-late pregnancy and lower gestational weight gain, women with early GDM showed higher rates of hyperglycemia and pre-diabetes/diabetes at 3 months postpartum. This is in line with prior studies demonstrating risk of dysglycemia in the postpartum period following an early GDM diagnosis [4, 16, 17], including higher risk of type 2 diabetes within 3 years [17]. These findings highlight the importance of postpartum monitoring and intervention particularly in women diagnosed with early GDM.

In conclusion, our findings indicate that early GDM is characterized by more severe underlying hyperglycemia compared to standard GDM. Timely treatment of GDM and reduced gestational weight gain may attenuate increased perinatal risks associated with early GDM.

## Supplementary Information

Below is the link to the electronic supplementary material.

Supplementary Material 1

## Data Availability

The datasets generated during and/or analyzed in the current study are available from the corresponding author upon reasonable request. For the purpose of open access, the author has applied a Creative Commons Attribution (CC BY) license to any Author Accepted Manuscript version arising from this submission.

## References

[1] American Diabetes Association Professional Practice Committee, 2. Classification and diagnosis of diabetes: standards of medical care in Diabetes—2022. Diabetes Care. **45**, S17–S38 (2021) 10.2337/dc22-S002 34964875

[2] Diabetes in pregnancy: Management of diabetes and its complications from preconception to the postnatal period [article online], 2015. Available from https://www.nice.org.uk/guidance/ng3 25950069

[3] S. Riskin-Mashiah et al., First-Trimester fasting hyperglycemia and adverse pregnancy outcomes. Diabetes Care. **32**(9), 1639–1643 (2009) 19549728 10.2337/dc09-0688 PMC2732138

[4] A.N. Sweeting et al., Gestational diabetes mellitus in early pregnancy: evidence for poor pregnancy outcomes despite treatment. Diabetes Care. **39**(1), 75–81 (2015) 26645084 10.2337/dc15-0433

[5] M.N. Feghali et al., Pregnancy outcomes in women with an early diagnosis of gestational diabetes mellitus. Diabetes Res. Clin. Pract. **138**, 177–186 (2018) 29427694 10.1016/j.diabres.2018.02.004 PMC5910191

[6] B.E. Metzger et al., Hyperglycemia and adverse pregnancy outcomes. N Engl. J. Med. **358**(19), 1991–2002 (2008) 18463375 10.1056/NEJMoa0707943

[7] D. Simmons et al., Treatment of gestational diabetes mellitus diagnosed early in pregnancy. N Engl. J. Med. **388**(23), 2132–2144 (2023) 37144983 10.1056/NEJMoa2214956

[8] C.N. Seifu et al., Association between immediate treatment of early gestational diabetes mellitus and breastfeeding outcomes: findings from the TOBOGM study. Diabetes Care. **47**(12), e99–e101 (2024) 38441605 10.2337/dc23-1635

[9] T. Thaweethai et al., Distinct insulin physiology trajectories in euglycemic pregnancy and gestational diabetes mellitus. Diabetes Care. **46**(12), 2137–2146 (2023) 37126832 10.2337/dc22-2226 PMC10698215

[10] L. Bozkurt et al., Pathophysiological characteristics and effects of obesity in women with early and late manifestation of gestational diabetes diagnosed by the international association of diabetes and pregnancy study groups criteria. J. Clin. Endocrinol. Metabolism. **100**(3), 1113–1120 (2015) 10.1210/jc.2014-4055 PMC4333043 25574889

[11] L.C. Kusinski, D.L. Jones, N. Atta, E. Turner, S. Smith, L.M. Oude Griep, K.L. Rennie, De E. Lucia Rolfe, S.J. Sharp, V. Farewell, H.R. Murphy, R. Taylor, C.L. Meek, Reduced energy diet in women with gestational diabetes: the dietary intervention in gestational diabetes DiGest randomised clinical trial. Nature Medicine, In Press 10.1038/s41591-024-03356-1 PMC11839452 39972237

[12] L.C. Kusinski et al., Dietary intervention in pregnant women with gestational Diabetes; protocol for the digest randomised controlled trial. Nutrients, 2020. **12**(4) 10.3390/nu12041165 PMC7230897 32331244

[13] S. Thangaratinam et al., *Guidance for Maternal Medicine Services in the Coronavirus (COVID-19) Pandemic* (Royal College of Obstetricians and Gynaecologists (ed), 2020)

[14] D.L. Jones et al., Dietary intakes and physical activity of women with gestational diabetes are not associated with continuous glucose monitoring metrics; secondary analysis of the digest trial. Clin. Nutr. ESPEN. **70**, 29–35 (2025) 40812416 10.1016/j.clnesp.2025.08.007

[15] D. Simmons et al., Perinatal outcomes in early and late gestational diabetes mellitus after treatment from 24–28 weeks’ gestation: A TOBOGM secondary analysis. Diabetes Care. **47**(12), 2093–2101 (2024) 38421672 10.2337/dc23-1667

[16] M.L. Champion et al., Postpartum glucose intolerance following early gestational diabetes mellitus. Am. J. Obstet. Gynecol. MFM. **4**(3), 100609 (2022) 35272093 10.1016/j.ajogmf.2022.100609 PMC9195159

[17] J.A. Svare, B.B. Hansen, L. Molsted-Pedersen, Perinatal complications in women with gestational diabetes mellitus. Acta Obstet. Gynecol. Scand. **80**(10), 899–904 (2001) 11580734 10.1034/j.1600-0412.2001.801006.x

