# Supplementary material for: The association between early diagnosis of gestational diabetes and maternal-neonatal outcomes: a secondary analysis of the digest trial
Source: Endocrine. 2026 Feb 16;91(1):87. doi: 10.1007/s12020-025-04542-y (PMC12909440; doi:10.1007/s12020-025-04542-y)
Supplement: Supplementary file 1 — Supplementary Material 1 [file 12020_2025_4542_MOESM1_ESM.docx]

**Table 1.** Baseline characteristics of participants enrolled in the DiGest trial and with information about timing of diagnosis.

|  | **n** | **All participants (n=417)** | **n** | **Early diagnosis (n=118)** | **n** | **Standard diagnosis (n=299)** | **p-value** |  |
| --- | --- | --- | --- | --- | --- | --- | --- | --- |
| **Gestational weeks at diagnosis** | | 417 | 22.89±6.39 | 118 | 13.69±3.38 | 299 | 26.52±2.41 | **<0.001** |
| **Maternal age (year)** | 417 | 33.02±5.04 | 118 | 33.65±4.88 | 299 | 32.78±5.08 | 0.110 |  |
| **Maternal education (≥degree)** | 417 | 195 (46.76) | 118 | 49 (41.5) | 299 | 146 (48.8) | 0.178 |  |
| **Index of multiple deprivation decile** | 402 | 6.52±2.48 | 116 | 6.48±2.51 | 286 | 6.53±2.47 | 0.859 |  |
| **Primiparous** | 376 | 135 (35.90) | 114 | 20 (17.5) | 262 | 115 (43.9) | **<0.001** |  |
| **Ethnicity** | 417 |  | 118 |  | 299 |  | 0.608 |  |
| **White** |  | 329 (78.90) |  | 96 (81.4) |  | 233 (77.9) |  |  |
| **Asian** |  | 69 (16.55) |  | 17 (14.4) |  | 52 (17.4) |  |  |
| **Black** |  | 16 (3.84) |  | 5 (4.2) |  | 11 (3.7) |  |  |
| **Other** |  | 3 (0.72) |  | 0 (0.0) |  | 3 (1.0) |  |  |
| **Smoking and vape** | 414 | 45 (10.87) | 117 | 20 (17.1) | 297 | 25 (8.4) | **0.011** |  |
| **Previous GDM** | 416 | 116 (27.88) | 117 | 67 (57.3) | 299 | 49 (16.4) | **<0.001** |  |
|  |  |  |  |  |  |  |  |  |
| **BMI Kg/m^2^ at Booking** | 376 | 34.15±6.52 | 114 | 35.55±6.98 | 262 | 33.54±6.23 | **0.009** |  |
| **BMI Kg/m^2^ at Baseline** | 417 | 35.78 ± 6.42 | 118 | 36.18 ± 6.72 | 299 | 35.62 ± 6.30 | 0.415 |  |
| **Weight Gain Baseline-Booking (kg)** | 416 | 4.05 ± 5.95 | 118 | 2.17 ± 5.62 | 298 | 4.80 ± 5.93 | **<0.001** |  |
|  |  |  |  |  |  |  |  |  |
|  |  |  |  |  |  |  |  |  |
| **Glycaemia at Baseline (~29 weeks)** |  |  |  |  |  |  |  |  |
| **Mean CGM glucose mg/dL** | 352 | 103.90±13.95 | 99 | 104.57±12.60 | 253 | 103.64±14.46 | 0.575 |  |
| **TIR (63-120 mg/dL)%** | 352 | 77.10±18.45 | 99 | 76.81±17.46 | 253 | 77.21±18.86 | 0.854 |  |
| **TAR (63-120 mg/dL)%** | 352 | 21.25±19.22 | 99 | 22.81±17.94 | 253 | 21.04±19.73 | 0.735 |  |
| **TBR (63-120 mg/dL)%** | 352 | 1.65±2.93 | 99 | 1.38±2.07 | 253 | 1.76±3.20 | 0.285 |  |
| **TIR (63-140 mg/dL)%** | 352 | 90.80±11.06 | 99 | 90.35±10.72 | 253 | 90.98±11.21 | 0.633 |  |
| **TAR (63-140 mg/dL)%** | 352 | 7.54±11.38 | 99 | 8.26±10.99 | 253 | 7.26±11.53 | 0.460 |  |
| **TBR (63-140 mg/dL)%** | 352 | 1.65±2.93 | 99 | 1.38±2.07 | 253 | 1.76±3.20 | 0.285 |  |
| **SD** | 352 | 1.05±0.29 | 99 | 1.10±0.30 | 253 | 1.03±0.28 | 0.079 |  |
| **CV** | 352 | 18.18±3.86 | 99 | 18.79±4.25 | 253 | 17.95±3.68 | 0.085 |  |
| **HbA1c mmol/mol** | 147 | 39.00±4.63 | 58 | 40.12±4.19 | 89 | 38.27±4.77 | **0.017** |  |
| **Medication Use (Baseline)** |  |  |  |  |  |  |  |  |
| **Metformin** | 417 | 90 (21.58) | 118 | 38 (32.2) | 299 | 52 (17.4) | **<0.001** |  |
| **Long-acting insulin** | 417 | 98 (23.50) | 118 | 52 (44.1) | 299 | 46 (15.4) | **<0.001** |  |
| **Short-acting insulin** | 417 | 37 (8.87) | 118 | 19 (16.1) | 299 | 18 (6.0) | **0.001** |  |
| **Assigned to DiGest Diet** | 414 | 210 (50.7) | 118 | 57 (48.3) | 296 | 153 (51.7) | 0.534 |  |

Results are presented as mean ± SD or n (%). The difference between groups were assessed by t-test and Chi-square test with p-values reported. GDM: gestational diabetes mellitus; CGM: continuous glucose monitoring; TIR: time in range; TAR: time above range; TBR: time below range; SD: standard deviation; CV: coefficient of variation. 63-120 mg/dL is equivalent to 3.5-6.7 mmol/L whereas 63-140 mg/dL is equivalent to 3.5-7.8 mmol/L. Baseline: 28-30 weeks gestation.

**Table 2. Intervention (reduced-energy DiGest diet) effects on DiGest trial primary maternal and neonatal outcomes, adjusted for timing of diagnosis**

|  | **Early Diagnosis** | | **Standard Diagnosis** | | **Model 1** | | **Model 2** | |
| --- | --- | --- | --- | --- | --- | --- | --- | --- |
|  | **Intervention**  **(n=53)** | **Control**  **(n=60)** | **Intervention (n=141)** | **Control**  **(n=125)** |  |  |  | |
|  |  |  |  |  | **B (95% CI)** | **P** | **B (95% CI)** | **P** |
| **Maternal weight change (36 Weeks – Baseline; kg)** | -0.04 ± 4.43 | 0.76 ± 4.25 | 0.48 ± 4.16 | 0.41 ± 4.22 | -0.17 (-1.01, 0.66) | 0.684 | -0.21 (-1.07, 0.64) | 0.618 |
| **Neonatal birthweight z-score** | 0.56 ± 1.17 | 0.52 ± 0.88 | 0.45 ± 0.96 | 0.42 ± .0.93 | 0.02 (-0.18, 0.21) | 0.875 | 0.04 (-0.16, 0.23) | 0.706 |

Birthweight z-score was adjusted for gestation at birth and sex using INTERGROWTH birthweight standard. Baseline: 28-30 weeks gestation. Model 1: Linear regression model for the effects of the trial assignment (reduced-energy diet vs standard-energy diet) on maternal weight change and neonatal birthweight z-score, adjusted for weight at booking and study center. Model 2: Similar to Model 1 but additionally adjusted for timing of diagnosis (early vs standard GDM).
